# Correction: The impact of COVID-19 pandemic on key performance indicators in three Saudi hospitals

**DOI:** 10.1371/journal.pone.0294487

**Published:** 2023-11-10

**Authors:** Abeer Alharbi, Ranya S. Almana, Mohammed Aljuaid

There are errors in the Funding section. The correct Funding statement is: Initials of the authors who received each award. AA, RM, MA Grant numbers awarded to each author. Funded through the Researcher Supporting Project (RSP2023R481), King Saud University, Riyadh, Saudi Arabia. The full name of each funder. Funded through the Researcher Supporting Project (RSP2023R481), King Saud University, Riyadh, Saudi Arabia. URL of each funder website. https://dsrs.ksu.edu.sa/en Did the sponsors or funders play any role in the study design, data collection and analysis, decision to publish, or preparation of the manuscript? NO—The funders had no role in study design, data collection and analysis, decision to publish, or preparation of the manuscript.
