# Supplementary material for: The impact of COVID-19 pandemic on key performance indicators in three Saudi hospitals
Source: PLoS One. 2023 May 11;18(5):e0285616. doi: 10.1371/journal.pone.0285616 (PMC10174493; doi:10.1371/journal.pone.0285616)
Supplement: S1 Table — (DOCX) [file pone.0285616.s001.docx]

**Appendix 1**

Total number of OPD visits in each COVID-19 stage in each hospital

| Hospital | Pre COVID  (Sep 2019 to Feb 2020) | Early stage of COVID  (Mar to Jul 2020) | Late stage of COVID  (Aug 2020 to Dec 2021) |
| --- | --- | --- | --- |
| GEN | 89,468 | 13801 | 88938 |
| MCH | 80,135 | 12609 | 119997 |
| MDC | 271,968 | 25898 | 445523 |

Total number of OR visits in each COVID-19 stage in each hospital

| Hospital | Pre COVID  (Sep 2019 to Feb 2020) | Early stage of COVID  (Mar to Jul 2020) | Late stage of COVID  (Aug 2020 to Dec 2021) |
| --- | --- | --- | --- |
| GEN | 2794 | 238 | 2789 |
| MCH | 1643 | 972 | 3225 |
| MDC | 11369 | 5379 | 31718 |
