# Supplementary material for: The impact of COVID-19 pandemic on key performance indicators in three Saudi hospitals
Source: PLoS One. 2023 May 11;18(5):e0285616. doi: 10.1371/journal.pone.0285616 (PMC10174493; doi:10.1371/journal.pone.0285616)
Supplement: Attachment — Submitted filename: Response to Reviewers.docx [file pone.0285616.s003.docx]

**Response to Reviewers**

**Reviewer #1**

**Thank you so much for your valuable comments. Below are my responses for each point.**

**1.** Table 1 Data Collection, in the sample size category, the author wrote 100% without stating the number of sample size? Does the 100% is referring to the sample size obtained? Best if the author can state the number of sample obtained in frequency too. The sample size obtained for the study is unclear. Is the bed capacity in the respective hospitals is your actual sample?

For our research, we used secondary data from Adaa’ program, collected to measure hospital quality metrics. This data did not report the exact sample size but did explain that for the OPD indicator, the data collectors used 100% of the patient’s data who used the OPD during the study period. Similarly, for the OR indicator, the data collectors used 100% of the patient’s data who used the OR during the study period. However, we added the visits numbers for the OPD and OR during the study three stages (pre-COVID, early-COVID, and post-COVID) in the supplement file appendix 1.

**2.** Table 3 Analysis of Variance; best if the author can limit the decimal point to the two decimal point for standardization in statistic. Please follow journal requirement. If it is not stated, two decimal point is best used to indicate the statistic results.

Corrected as advised.

**3.** Paragraph for discussion (line 185 - 193) is not needed. Focus your discussion based on the result without stating facts that have been previously discussed earlier in the introduction section.

Deleted as advised.

**4.** The paper is lacking in critical recommendation on the issue, while the author is pushing for proposed contingency plan? what kind of contingency plan that should be proposed for the pandemic? This might add value to the policy maker. Thus the conclusion seemed lacking in providing concrete ending to what should be done to prevent/ address the issue.

Recommendations has been added to the conclusion section (Lines 254-260)

**Reviewer #2**

A well organized article. The ethical review for the research meets the required standards. The methods of statistical analysis used for the research were sound and the results of the 2 way ANOVA test supported the conclusion of the article. Enough data was provided in the article although it would have been easier to absorb/understand the provided data used for analysis (OPD waiting times in days and Elective OR utilization rates during Pre, early and post pandemic periods) if they were all included in a single table of data extraction. The sizes of figures 1-4 could be increased a bit to provide better visibility for the reader. The article was written intelligibly, with appropriate use of the English language although there unnecessary repetition of facts in the introduction and discussion sections eg ''To contain the spread of the COVID-19 disease, the Saudi government adopted the lockdown strategy''. Adequate explanation of the reasons for variation in the results/findings among the 3 hospitals were give in the discussion section with good interpretation of the findings. The limitations were addressed properly and the conclusion was brief and concise. In general, it was a very interesting and quite unique article as it aims to explore, compare and analyse the differences in pandemic effects on Key performance indicators in 3 hospitals providing very different types of medical care.

**Thank you so much for your valuable comments.**

The data used for analysis (OPD waiting times in days and Elective OR utilization rates during Pre, early and post pandemic periods) were all included in a supplemental file.

The figures have been increased in size for better visibility.

Also, the unnecessary repetition of facts in the discussion section has been deleted.
